# Metabolomics Study Revealed the Effects of CaO-Treated Maize Straw on the Rumen Metabolites

**DOI:** 10.3390/ani15050674

**Published:** 2025-02-26

**Authors:** Hui Wang, Mingjun Shi, Zhanxia Ma, Xuewei Zhang, Huiyong Shan, Xiaofeng Xu, Suyu Quan, Junqin Zhang, Yujia Tian

**Affiliations:** 1 Tianjin Key Laboratory of Agricultural Animal Breeding and Healthy Husbandry, College of Animal Science and Veterinary Medicine, Tianjin Agricultural University, Tianjin 300392, China; w1010045898@gmail.com (H.W.); 15978729616@163.com (M.S.); mazhanxia0607@163.com (Z.M.); zhangxuewei63@163.com (X.Z.); 2 College of Engineering and Technology, Tianjin Agricultural University, Tianjin 300392, China; tjshyyr@sina.com; 3 College of Animal Science and Technology, Ningxia University, Yinchuan 750021, China; xuxiaofengnd@126.com; 4 Forage Feed Workstation, Animal Husbandry Bureau of Fangshan Country, Lyuliang 033100, China; 15535838194@163.com

**Keywords:** CaO treatment, *in vitro* culture, maize straw, rumen metabolomics

## Abstract

As an important limiting factor, lignin hinders the utilization rate of maize straw in ruminants. CaO treatment increases the feed digestibility of maize straw by disrupting the ester bonds between hemicellulose, cellulose, and lignin in maize straw. Our previous research found that CaO treatment of corn straw may increase its feed digestibility by altering the rumen microbes’ abundance. This study further investigated the molecular mechanism of CaO treatment to enhance feed utilization and also examined its ongoing effects on rumen metabolites. Rumen fluid was collected to analyze microbial metabolites using liquid chromatography–mass spectrometry (LC–MS) non-targeted metabolomics. Maize straw (moisture content of 60%) treated with four levels of CaO (0%, 3%, 5%, and 7%) was used as the fermentation substrate for a 6 h *in vitro* culture. Based on the effect of CaO-treated maize straw on the rumen microbial diversity, no significant differences were observed in microbial composition between the 0% and 3% treatment groups or between the 5% and 7% treatment groups. However, the microbial structure of the 0% and 3% treatment groups differed from that of the 5% and 7% groups. Therefore, the four levels were divided into a low-efficiency group (LE group: 0% and 3% levels) and a high-efficiency group (HE group: 5% and 7% levels) for principal component analysis (PCA) and partial least squares discriminant analysis (PLS-DA). Compared with the LE group, most of the ruminal metabolites that showed increased levels in the HE group were products of lignin degradation. Among these differential metabolites, Dihydro-3-coumaric acid had a significant positive correlation with *Prevotella* and fermentation indicators like acetate, propionate, and butyrate. KEGG analysis showed differential metabolites were primarily enriched in the amino acid metabolism, tryptophan metabolism, phenylalanine, tyrosine, and tryptophan biosynthesis, and cyanoamino acid metabolism. The higher CaO concentration in the HE group effectively disrupted most covalent bonds with lignin, significantly enhancing cellulose degradation and ultimately supporting improved rumen metabolism.

## Simple Summary

Untreated maize straw has a low feed digestibility because of its high cellulose and lignin content, which restricts its use in animal feeding. A previous study by our team has demonstrated that calcium oxide (CaO), an affordable and eco-friendly alkaline reagent, may enhance the rumen degradation rate and fermentation effect of maize straw, while enriching the abundance of cellulose-degrading microorganisms. This study further explored the effects of different levels of CaO treatment on corn straw on rumen metabolism by liquid chromatography–mass spectrometry non-targeted metabolomics. Results showed that CaO in the HE group (High-Efficiency group: 5% and 7% levels) significantly broke down the network structure formed by lignin and cell wall polysaccharides in corn stalks, and improved cellulose metabolism and amino acid synthesis and metabolism. This study provides data for the efficient utilization of crop by-product maize straw as feed, contributing to the sustainable development of agriculture.

## 1. Introduction

Maize straw is rich in cellulose, hemicellulose, and lignin, making it a potential energy source for ruminants [1]. However, the network structure formed by lignin and cell wall polysaccharides reduces the degradability of straw cell walls, leading to low feed energy conversion efficiency when untreated maize straw is directly fed to ruminants [2,3]. Therefore, researchers continue to investigate various treatments for crop residues to enhance their efficient utilization by ruminants, with a focus on physical, chemical, and biological methods. Calcium oxide (CaO), an affordable and eco-friendly alkaline reagent, effectively disrupts the copolymer bonds between hemicellulose and lignin, releasing fermentable carbohydrates and enhancing forage digestibility in ruminants [4,5]. Ciriaco et al. [6] reported that treating hay with 5% CaO (DM basis) increased its *in vitro* degradation rate. Similarly, our previous research demonstrated that maize straw treated with 5% and 7% CaO (DM basis) enhanced its utilization by destroying the lignin structure [7]. Furthermore, this treatment influenced rumen microbial diversity, with a significant difference observed during the 6 h of *in vitro* fermentation [8]. However, the effects of CaO-treated maize straw on rumen microbial metabolism remain unexplored.

The establishment of microbial flora is essential for rumen metabolism [9]. A combined microbiome and metabolome analysis has been used to investigate the role of dietary nutrients in rumen microbial metabolism, providing insights into the effects of early nutritional interventions and plant extracts on rumen function [10,11]. Li et al. [11] reported that supplementation of dandelion enhanced rumen fermentation in lactating cows by promoting microbial degradation of structural carbohydrates and starch. Similarly, Ogunade et al. [12] found that monensin influenced rumen fermentation of forage-fed beef cattle by inhibiting the biohydrogenation of unsaturated fatty acids, and reducing linoleic acid and amino acid metabolism. Our research demonstrated that maize straw treated with CaO improved rumen fermentation and microbial diversity, potentially altering rumen microbial metabolism.

Our previous research demonstrated that CaO-treated maize straw could affect the abundance of rumen microorganisms, especially promoting the enrichment of *Prevotella_1*. In this study, we hypothesized that maize straw treated with CaO alters the abundance of key microorganisms, and further affects the metabolism of nutrients. To explore this, we used LC–MS non-targeted metabolomics to analyze the effects of CaO-treated straw on key markers and pathways in rumen metabolism. Our findings aim to provide mechanistic insights into the metabolic processes of CaO-treated maize straw in the rumen.

## 2. Materials and Methods

### 2.1. Experimental Design

Maize straw (variety: Yufeng 303) was randomly collected from five one-square-meter plots in Hebei province, China (36°05′–42°40′ N, 113°27′–119°50′ E). The collected maize straw was mixed and all cut into pieces of 2–3 cm length. The straw (moisture content was adjusted to 60%) was treated with four different levels of CaO (0%, 3%, 5%, and 7% of dry straw weight). Each treatment had 12 replicates, totaling 24 samples. Based on our previous study, no significant differences in the microbial structure were observed between the 0% and 3% treatment groups or between the 5% and 7% treatment groups. However, the microbial structure of the 0% and 3% groups differed from that of the 5% and 7% groups. Accordingly, samples treated with 0% and 3% CaO were considered the low-efficiency group (LE group), while those treated with 5% and 7% CaO were regarded as the high-efficiency (HE group). All straws were fermented in 1 L anaerobic fermentation buckets for 30 days. After fermentation, straws were placed in a constant temperature air oven at 65 °C to dry for 48 h. Air-dried maize straws were crushed to 0.25 mm and put into plastic bags for further processing.

### 2.2. Ruminal Inoculum and In Vitro Rumen Fermentation

In the *in vitro* study, ruminal fluid was obtained from four non-pregnant Holstein heifers (body weight = 610 ± 20 kg) fitted with rumen cannulae. The heifers were fed according to Nation Research Council (NRC) 1.3 maintenance nutrients levels (Table 1). Before feeding, ruminal fluid was collected via rumen fistulas, thoroughly mixed, and immediately transported to the laboratory. Under the condition of a 39 °C water bath, the rumen fluid was filtered with four-layer cheesecloth, and CO_2_ was introduced while filtering to ensure an anaerobic environment.

*In vitro* batch fermentation was performed in twenty-four 100 mL serum bottles using CaO-treated corn straw as the fermentation substrate. The fermentation buffer medium was prepared according to the method described by Menke et al. [13]. Each serum bottle, under anaerobic conditions, was filled with 1 g of the substrate and 75 mL of artificial rumen fluid (filtered rumen fluid to fermentation buffer ratio of 1:2). All serum bottles were immediately placed in a constant temperature water bath oscillator with an oscillation frequency of 45 r/min at 39 °C for 6 h.

**Table 1:** Ingredients and chemical composition of the diet fed to the donor cattle.

Total Mixed Ration	
Ingredient, g/kg DM	
Corn meal	230
Alfalfa hay	449
Chinese wild rye hay	150
Wheat bran	42
Soybean meal	52
Cottonseed meal	64
Dicalcium phosphate	4.2
NaCl	4.2
Premix ^(1)^	4.2
**Chemical composition**	
DM, g/kg	527.5
CP, g/kg DM	158
aNDF, g/kg DM	432
ADF, g/kg DM	295
NE_L_, Mcal/kg *	1.4

^(1)^ Contained 10% Mg; 5000 mg/kg of Zn; 1000 mg/kg of Mn; 2500 mg/kg of Fe; 2600 mg/kg of Cu; 192 mg/kg of I; 45 mg/kg of Co; 60 mg/kg of Se; 1,240,000 IU/kg of vitamin A; 50,000 IU/kg of vitamin D; 10,500 IU/kg of vitamin E. DM, dry matter; NE_L_, net energy for lactation; CP, crude protein; aNDF, neutral detergent fiber analyzed with a heat-stable amylase; ADF, acid detergent fiber. * According to NRC (2001) [14].

### 2.3. Metabolomics Analysis

100 μL *in vitro* fermentation fluid and 400 μL of extraction solution were mixed in a 1.5 mL centrifuge tube. The extraction solution consisted of a 1:1 (*v*/*v*) mixture of acetonitrile and methanol, containing 0.02 mg/mL of L-2-chlorophenylalanine. The samples were vortexed for 30 s to mix evenly, followed by sonication for 30 min at a temperature of 5 °C and a frequency of 40 KHz, then the samples were left to stand for 30 min at −20 °C. After the proteins were completely precipitated, the samples were centrifuged at 13,000× *g* for 15 min at 4 °C, and the supernatant was collected and evaporated to dryness under a stream of nitrogen gas. A mixed solution of acetonitrile and water at a 1:1 ratio was prepared, and the dried sample was redissolved in 100 µL of the mixed solution. The reconstituted solution underwent low temperature ultrasonication for 5 min, followed by centrifugation for 10 min. The final supernatant was for subsequent LC–MS analysis.

The LC–MS/MS analysis was provided by Majorbio Bio-Pharm Technology Co., Ltd. (Shanghai, China).

### 2.4. Data Analysis

In the present study, the PCA and PLS-DA were performed using Majorbio Cloud Platform (https://www.majorbio.com/, accessed on 4 August 2023). Differentially expressed metabolites were selected according to VIP (Variable Importance Projection) > 1 and statistical analysis (*p* < 0.05). The selected differentially expressed metabolites were analyzed using scipy in Python (Version 1.0.0) for the pathway enrichment analysis. Metabolite relationship analysis was conducted using Pearson’s correlation algorithm.

## 3. Results

### 3.1. Principal Component Analysis of Microbial Metabolites

**Figure 1:**
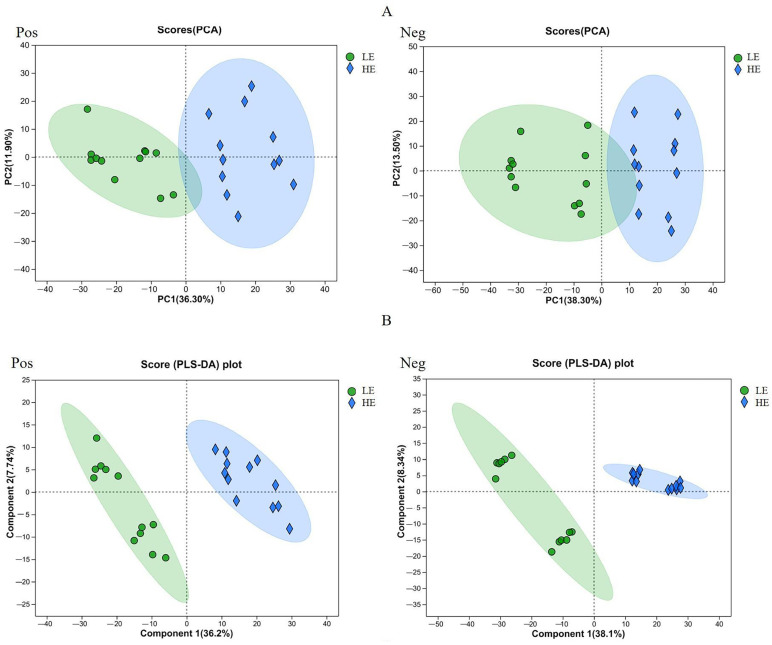
Ruminal metabolomic profile of straw treatment with the high-efficiency CaO (HE group; solid blue circles) and the low-efficiency CaO (LE group; green circles). (**A**) Principal component analysis (PCA); (**B**) Partial least squares discriminant analysis (PLS-DA), horizontal axis (component 1), the first principal component, vertical axis (component 2), the second principal component.

The PCA results showed that the HE and LE groups had some similar metabolites (Figure 1A). However, PLS-DA analysis (Figure 1B) showed that the HE group samples were positioned on the right side of the longitudinal axis, distinctly separated from the LE group, indicating that different CaO treatments led to significant differences in rumen metabolites. All samples were within the 95% confidence interval, with no outliers detected, confirming the stability and repeatability of the analysis.

### 3.2. Differential Metabolite Analysis

**Figure 2:**
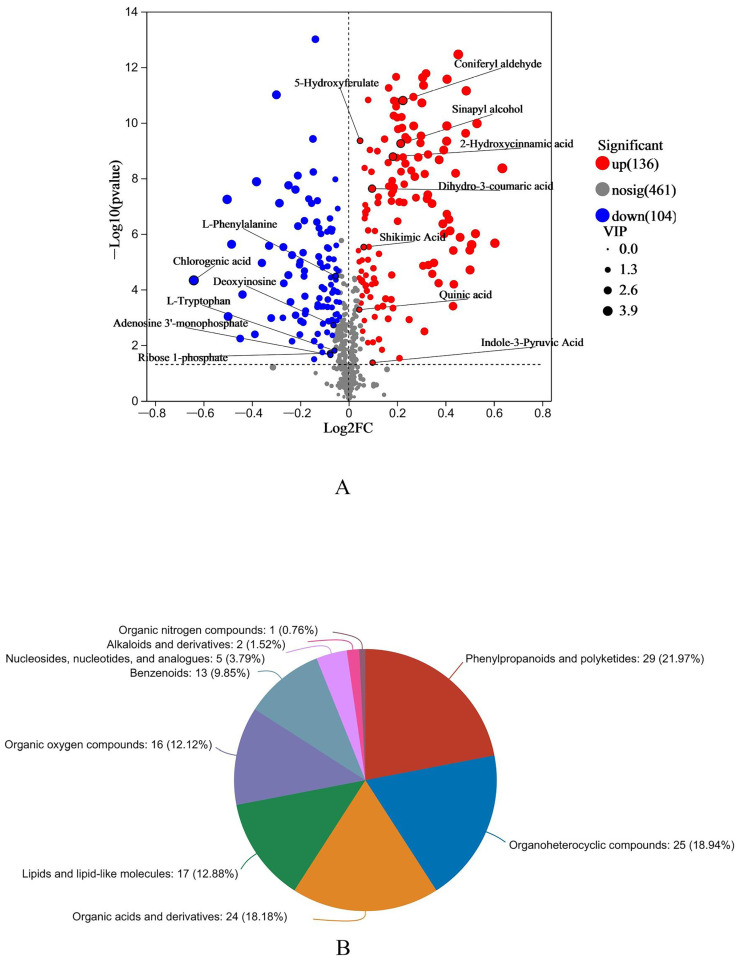
(**A**) Volcano plots; HE group vs the LE group. (**B**) Pie chart of the different metabolites between the HE group and LE group in HMDB chemical classification (superclass).

Based on OPLS-DA with VIP > 1, and *t*-test with *p* < 0.05, significantly differential metabolites between the two groups were screened. A total of 240 differential metabolites (136 upregulated and 104 downregulated) were detected in the HE group compared with the LE group (Figure 2A). Each point represents a metabolite, and all differential metabolites obtained from the LE group and HE group were matched with the HMDB database (https://www.hmdb.ca/, accessed on 4 August 2023). Figure 2B shows the HMBD classifications of the top 20 metabolites. The main classes of differential metabolites were phenylpropanoids and polyketides (29 metabolites, 21.97%), organoheterocyclic compounds (25 metabolites, 18.94%), organic acids and derivatives (24 metabolites, 18.18%), lipids and lipid-like molecules (17 metabolites, 12.88%), and organic oxygen compounds (16 metabolites, 12.12%).

### 3.3. KEGG Enrichment Analysis

**Figure 3:**
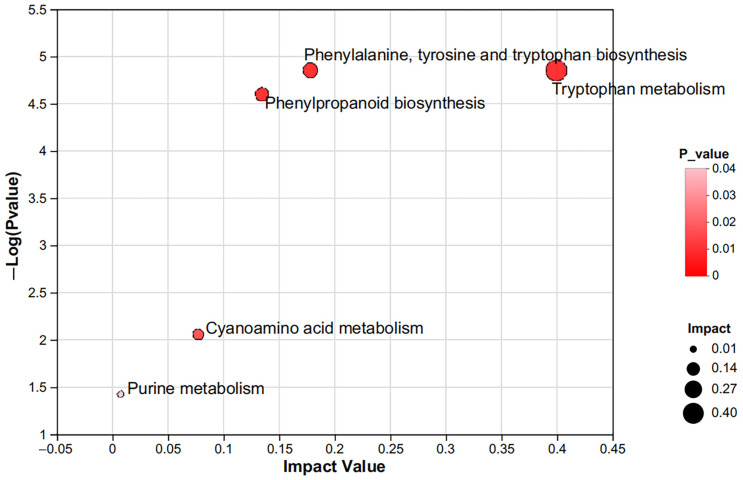
KEGG pathway enrichment analysis. Each bubble in the figure represents a KEGG pathway. The horizontal axis indicates pathway impact value, and the vertical axis indicates *p* value of pathway; a darker color indicates smaller *p* values, a larger size of bubble indicates more metabolites enriched in the pathway.

According to the screened differently expressed metabolites, KEGG analysis was performed (Figure 3). The differently expressed metabolites were primarily enriched in five metabolic pathways: tryptophan metabolism, phenylalanine, tyrosine, tryptophan biosynthesis, phenylpropanoid biosynthesis, cyanoamino acid metabolism, and purine metabolism. Differential metabolites involved in tryptophan metabolism included 5-hydroxy-L-tryptophan, L-kynurenine, 3-indoleacetic acid, L-tryptophan, indole-3-pyruvic acid, and 5-hydroxyindoleacetic acid. The key metabolites associated with these metabolic pathways are listed in Table 2.

### 3.4. The Analysis of Correlation Between Fermentation Parameters and Differential Metabolites

**Figure 4:**
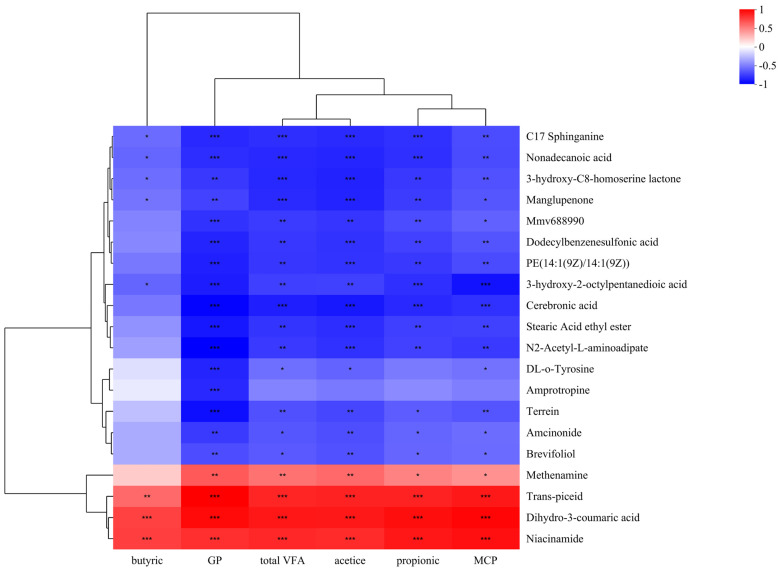
Correlation analysis between rumen fermentation and metabolites. * 0.01 < *p* ≤ 0.05; ** 0.001 < *p* ≤ 0.01; *** *p* ≤ 0.001.

To identify metabolites related to rumen fermentation, Pearson correlation analysis was conducted among rumen fermentation indicators, MCP, and filtrated differential metabolites. As shown in the correlation heatmap (Figure 4), niacinamide, dihydro-3-coumaric acid, and trans-piceid exhibited positive correlations with acetic acid, propionic acid, butyric acid, total volatile fatty acids (VFA), gas production, and rumen MCP. A slice of metabolites was negatively correlated with these indicators, such as 3-hydroxy-2-octylpentanedioic acid, manglupenone, 3-hydroxy-C8-homoserine lactone, nonadecanoic acid, C17 sphinganine.

### 3.5. Correlation Analysis Between Rumen Microbial Composition and Differential Metabolites

**Figure 5:**
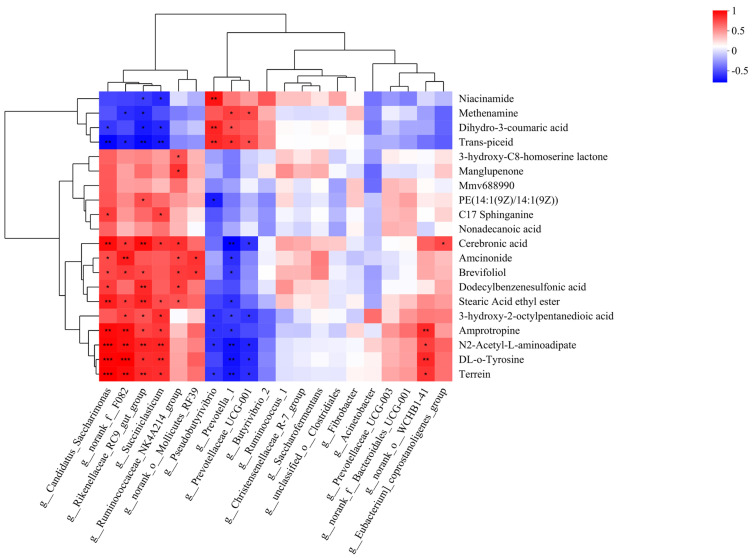
Correlation analysis between microbiota and metabolites. * 0.01 < *p* ≤ 0.05; ** 0.001 < *p* ≤ 0.01; *** *p* ≤ 0.001.

The correlation heat map is shown in Figure 5. The abundance of *Prevotella_1* was positively correlated with dihydro-3-coumaric acid, methenamine, and trans-piceid levels, and negatively associated with Stearic acid ethyl ester, DL-o-Tyrosine, N2-Acetyl-L-aminoadipate, Amprotropine, Terrein, 3-hydroxy-2-octylpentanedioic acid, Cerebronic acid, Amcinonide, and Brevifolio levels.

## 4. Discussion

In the present study, metabolomics analyses of rumen fluid based on LC–MS non-targeted metabolomics revealed the effect of CaO-treated corn straw on the ruminal metabolites. PCA and PLS-DA analyses indicated differences in ruminal metabolites for different concentrations of CaO treatment with corn straw. Notably, “Phenylpropanoids and polyketides” emerged as the most abundant metabolite category, with the HE group showing significant enrichment in this class (28 out of 136 upregulated metabolites). The substantial increase in these metabolites suggests that the lignocellulose structure of maize straw in the HE group was more extensively disrupted, leading to a greater accessible surface area and enhanced release of lignin fragments. Consequently, lignin degradation was facilitated, promoting the production and accumulation of lignin-derived monomers, including flavonoids and hydroxystilbenes [15].

Correlation analysis between metabolites and rumen microbiome revealed a positive association between dihydro-3-coumaric acid and *Prevotella_1*. Dihydro-3-coumaric acid (3-Hydroxyphenylpropionic acid) was significantly upregulated in the HE group and is derived from the metabolism of *p*-coumaric and ferulic acids [16]. These hydroxycinnamic acids are rarely found in the free acid form in all plants; they are usually covalently linked to lignin and/or hemicellulose via ester bonds and/or ether bonds. Most ferulic acid is ether-linked to lignin and simultaneously ester-linked to polysaccharides [17,18,19]. Studies have shown that mild alkaline treatment promoted the cleavage of ester bonds between lignin and hemicellulose, leading to the release of esterified ferulic acid and coumaric acid [20,21,22]. *Prevotella* produced feruloyl esterase (FAE), an enzyme capable of cleaving ester bonds between ferulic acid and attached sugars [23], thereby releasing free ferulic acid and polysaccharides (cellulose, hemicellulose, and pectin), which serve as substrates for bacteria, fungi, and protozoa in the rumen. Under the action of rumen microorganisms, ferulic acids produced 3-Hydroxyphenylpropionic acid through side chain hydrogenation and C4 dehydroxylation [24]. The further degradation of polysaccharides in the rumen results in the production of volatile fatty acids (VFAs), explaining the significant positive correlation between dihydro-3-coumaric acid and fermentation parameters such as acetic, propionic, and butyric. In addition, our research team previously found that *Prevotella* was rapidly enriched in the HE group, accompanied by a significant increase in acetate, propionate, and butyrate levels [7]. Taken together, these findings suggest that in the HE group, the cleavage of covalent bonds in maize straw cell walls facilitated the release of fermentable substrates, promoting *Prevotella* enrichment. This, in turn, enhanced FAE production, accelerating cellulose metabolism and nutrient release. Collectively, these results showed that cellulose metabolism in the rumen was effectively promoted in the HE group.

We explored the key metabolic pathways based on impact value and *p* value [25]. KEGG analysis showed that amino acid metabolism was enriched in the rumen of the HE group, including tryptophan metabolism, phenylalanine, tyrosine, and tryptophan biosynthesis, as well as cyanoamino acid metabolism.

Tyrosine, phenylalanine, and tryptophan are aromatic amino acids (AAA) essential for protein synthesis, with their biosynthetic pathways occurring in bacteria, fungi, and some protozoa. Shikimic acid and quinic acid serve as organic acid precursors of AAA biosynthesis [26]. In the HE group, these upstream metabolites increased, whereas phenylalanine and tryptophan contents decreased. As phenylalanine is a precursor in the phenylpropanoid biosynthesis pathway, its depletion corresponded with a significant enrichment of this pathway, leading to the synthesis of coniferyl aldehyde, sinapyl alcohol, 5-hydroxyferulate, 2-hydroxycinnamic acid, and chlorogenic acid. Chlorogenic acid was hydrolyzed by microbial esterase to produce quinic acid, which can further participate in phenylalanine, tyrosine, and tryptophan biosynthesis. Additionally, phenylalanine plays a role in cyanoamino acid metabolism, a pathway involved in cyanide detoxification [27], thereby reducing the adverse effects of cyanide poisoning in ruminants. Previous studies have reported a positive correlation between the abundance of *Prevotella* species and cyanoamino acid metabolism [28]. We hypothesize that CaO-treated maize straw promoted the enrichment of *Prevotella* in the rumen, thereby enhancing cyanoamino acid metabolisms. As an essential amino acid, tryptophan undergoes further metabolism to generate bioactive compounds such as serotonin, niacin, and melatonin. Indole-3-pyruvic acid, a key product of tryptophan metabolism, was significantly upregulated in the HE group, potentially contributing to improved gut–blood barrier function, protection against oxidative stress, and attenuation of inflammation [29].

Furthermore, the concentrations of adenosine 3′-monophosphate, deoxyinosine, and ribose 1-phosphate changed significantly in this study, indicating alterations in the purine metabolism pathway. Enhanced purine metabolism is associated with increased microbial protein synthesis in the rumen [30]. Correspondingly, several studies have reported that alkali-treated straw increased the content of MCP [31,32,33]. Therefore, CaO-treated corn straw in the HE group improved purine metabolism by increasing the content of rumen MCP.

## 5. Conclusions

Compared with the LE group, maize straw treated with high levels of CaO significantly enhanced lignocellulose degradation, facilitated cellulose utilization by rumen microorganisms, and improved amino acid and purine metabolism. Additionally, the HE group exhibited an increase in potentially beneficial metabolites, such as cinnamic acids and their derivatives. These findings highlight the potential of high-concentration CaO treatment to improve the digestibility of high-fiber forages and its broader implications for optimizing rumen metabolism.

## Figures and Tables

**Table 2:** Differential metabolites in the key metabolic pathways.

Pathway	Metabolites
Tryptophan metabolism	5-Hydroxy-L-tryptophan, L-Kynurenine, 3-Indoleacetic Acid, L-Tryptophan, Indole-3-Pyruvic Acid, 5-Hydroxyindoleacetic acid
Phenylalanine, tyrosine, and tryptophan biosynthesis	L-Phenylalanine, L-Tryptophan, Shikimic Acid, 5-Dehydroquinic acid, Quinic acid
Phenylpropanoid biosynthesis	Coniferyl aldehyde, L-Phenylalanine, Sinapyl alcohol, Chlorogenic acid, 5-Hydroxyferulate, 2-Hydroxycinnamic acid
Cyanoamino acid metabolism	N,N-Dihydroxy-L-tyrosine, L-Phenylalanine, N,N-Dihydroxy-L-phenylalanine
Purine metabolism	Adenosine 3′-monophosphate, Deoxyinosine, Ribose 1-phosphate

## Data Availability

The original contributions presented in this study are included in the article/Supplementary Material; further inquiries can be directed to the corresponding author.

## Supplementary Materials

The following supporting information can be downloaded at: https://www.mdpi.com/article/10.3390/ani15050674/s1, Table S1: Fermentation parameters and rumen microbial protein content for each group; Table S2: Rumen microbial composition for each group.
